# Novel Oxytocin Gene Expression in the Hindbrain Is Induced by Alcohol Exposure: Transgenic Zebrafish Enable Visualization of Sensitive Neurons

**DOI:** 10.1371/journal.pone.0053991

**Published:** 2013-01-14

**Authors:** Caitrín M. Coffey, Patricia A. Solleveld, Joyce Fang, Antonia K. Roberts, Sung-Kook Hong, Igor B. Dawid, Caroline E. Laverriere, Eric Glasgow

**Affiliations:** 1 Department of Oncology, Lombardi Comprehensive Cancer Center, Georgetown University Medical Center, Washington, District of Columbia, United States of America; 2 Laboratory of Molecular Genetics, Program in Genomics of Differentiation, Eunice Kennedy Shriver National Institute of Child and Human Development, National Institutes of Health, Bethesda, Maryland, United States of America; 3 Molecular Genetics Branch, National Human Genome Research Institute, National Institutes of Health, Bethesda, Maryland, United States of America; Institute of Cellular and Organismic Biology, Taiwan

## Abstract

**Background:**

Fetal Alcohol Spectrum Disorders (FASD) are a collection of disorders resulting from fetal ethanol exposure, which causes a wide range of physical, neurological and behavioral deficits including heightened susceptibility for alcoholism and addictive disorders. While a number of mechanisms have been proposed for how ethanol exposure disrupts brain development, with selective groups of neurons undergoing reduced proliferation, dysfunction and death, the induction of a new neurotransmitter phenotype by ethanol exposure has not yet been reported.

**Principal Findings:**

The effects of embryonic and larval ethanol exposure on brain development were visually monitored using transgenic zebrafish expressing cell-specific green fluorescent protein (GFP) marker genes. Specific subsets of GFP-expressing neurons were highly sensitive to ethanol exposure, but only during defined developmental windows. In the *med12* mutant, which affects the Mediator co-activator complex component Med12, exposure to lower concentrations of ethanol was sufficient to reduce GFP expression in transgenic embryos. In transgenic embryos and larva containing GFP driven by an *oxytocin-like* (*oxtl*) promoter, ethanol exposure dramatically up-regulated GFP expression in a small group of hindbrain neurons, while having no effect on expression in the neuroendocrine preoptic area.

**Conclusions:**

Alcohol exposure during limited embryonic periods impedes the development of specific, identifiable groups of neurons, and the *med12* mutation sensitizes these neurons to the deleterious effects of ethanol. In contrast, ethanol exposure induces *oxtl* expression in the hindbrain, a finding with profound implications for understanding alcoholism and other addictive disorders.

## Introduction

Fetal Alcohol Syndrome (FAS) is the most widely recognized consequence of prenatal alcohol exposure and is a leading cause of mental retardation [1]. FAS is characterized by three developmental anomalies: 1) prenatal and/or postnatal growth retardation; 2) a distinct facial appearance; and 3) central nervous system (CNS) dysfunction [2]. However, FAS is not the only result of prenatal alcohol exposure. Prenatal ethanol exposure results in a continuum of developmental anomalies leading to a spectrum of physical, mental and behavioral deficits, often with lifelong implications. The term Fetal Alcohol Spectrum Disorder (FASD) is used to describe these disorders. The total prevalence of FASD in the United States has been estimated to be as high as 2–5% [3]. In addition, low levels of fetal alcohol exposure may result in subtle neurodevelopmental consequences that are not necessarily identified as FASD, yet result in susceptibility to a range of neuropsychiatric and behavioral disorders including attention deficit hyperactivity disorder, depression, psychosis, drug addiction and alcoholism [2], [4]–[9].

An individual’s increased susceptibility to these neuropsychological and behavioral disorders is perhaps due in part to fetal ethanol exposure and to the individual’s genetic background. Genetics plays a significant role in an individual’s sensitivity to ethanol, and fetal exposure may exacerbate neurodevelopmental deficits resulting from certain genetic disorders [10], [11]. Genetic disorders associated with mutations in the *MED12* gene provide an interesting example. MED12, also known as TRAP230, is a component of Mediator, an evolutionarily conserved multi-protein complex that bridges gene regulatory regions with RNA polymerase, affecting the regulation of a large number of genes [12]. One potential example of fetal ethanol exposure influencing the prognosis of a genetic disorder is Opitz-Kaveggia syndrome, an X-linked mental retardation disorder resulting from mutations in MED12 [13]–[15]. Fetal alcohol exposure could aggravate the neuropsychiatric disorders associated with this syndrome, although the mechanisms involved are not currently understood.

Several hypotheses have been advanced for mechanisms by which fetal ethanol exposure leads to the reduced proliferation, neuronal dysfunction and cell death suggested to be causes of the clinically observed behavioral phenotype. These include: 1) depriving neurons of activity-dependent trophic factors by antagonizing NMDA receptors and potentiating GABAA receptors, thus, triggering apoptosis; 2) disruption of midline serotonergic neurons; 3) interference with L1 CAM function; 4) oxidative stress and free radical damage; 5) disruption of growth factor signaling (e.g., IGF); and 6) interference with neuronal metabolic enzymes [16]–[26]. The deleterious effect of ethanol on fetal brain development may proceed by any one or more of these mechanisms, or others yet to be discovered. In all scenarios, fetal alcohol exposure leads to reduced proliferation, neuronal dysfunction and/or cell death, with some groups of neurons being more sensitive to ethanol than others. The spectrum of mental and behavioral abnormalities associated with FASD is thought to arise as a result of this differential sensitivity, which could produce a pathogenic or suggestive phenotype for diagnosis [20].

Recently, improvements in imaging technologies have enabled investigators and clinicians to visualize structural changes in the brain, such as agenesis of the corpus callosum, thought to arise from high levels of fetal alcohol exposure [27], [28]. While these new technologies are exciting, higher resolution is required to answer critical questions regarding mechanisms and consequences of differential cellular responses to ethanol.

We are particularly interested in the potential effects of fetal alcohol exposure on the development of oxytocin expressing cells of the neuroendocrine hypothalamus, and the impact of oxytocin system dysfunction on the susceptibility to neurobehavioral disorders. This interest arises from the fact that the oxytocin system is known to mediate a wide range of social interactions, many of which are perturbed in individuals with FASD [2], [8], [29]–[33]. However, a direct link between the oxytocin system and FASD has not been established. Zebrafish are being utilized for these experiments because unlike other vertebrate models their anatomy and development allows for direct visualization of alterations in neuronal development in an organism that can be subsequently tested for resulting changes in behavior. The zebrafish *oxytocin-like* (*oxtl*) gene produces the neurohormone Isotocin, which is closely related to mammalian oxytocin [34]. Thus, we hypothesized that embryonic ethanol exposure would disrupt development of the *oxtl* system in zebrafish and lead to subsequent alterations in behavior.

In order to directly visualize the effect of ethanol exposure in intact developing brains at the cellular level, we utilized transgenic zebrafish that express green fluorescent protein (GFP) in specific subsets of neurons. Beyond their external fertilization and optical clarity, zebrafish present several additional experimental advantages for investigating perturbations of brain development. First, a large collection of transgenic lines that express fluorescent proteins in highly restricted neuronal populations has been generated by several laboratories [35]–[42]. Second, zebrafish are amenable to genetic analyses, and a number of mutant strains are readily available (http://zebrafish.org/zirc). Third, the larva and adults display a wide range of basal behaviors that are relevant for alcohol studies including memory, addiction and social behavior [43]–[47]. Finally, due to the small size of the zebrafish embryo, the entire nervous system can be observed as it develops.

In this study, four well-characterized transgenic lines were utilized to evaluate the hypothesis that specific subsets of neurons are sensitive to ethanol exposure. Our results demonstrate that zebrafish are well suited for visual detection of sensitive developmental periods of specific groups of ethanol-sensitive neurons. Accordingly, zebrafish provide an excellent model in which to comprehensively map ethanol dose, duration and stage of exposure to alterations in brain development at a cellular level. In addition, the effects of ethanol were evaluated in *med12^y82^* embryos, which carry a mutation in the transcriptional Mediator component, Med12 [48]. The results of these experiments suggest a highly productive new avenue for investigating the interaction between genetic factors and ethanol exposure. Finally, in order to test the idea that ethanol perturbs the development of the oxytocinergic system, we generated a transgenic line, which expresses GFP from the zebrafish *oxtl* promoter. Contrary to our expectations, ethanol treatment did not disrupt oxytocinergic system development but instead induced *oxtl* expression in the posterior hindbrain.

## Results

### Transgenic Zebrafish Reveal Neuronal Populations that are Particularly Sensitive to Ethanol Treatment

Four transgenic zebrafish lines expressing GFP in specific subsets of neurons were used to directly visualize the effect of ethanol exposure in intact developing brains.

**Figure 1:**
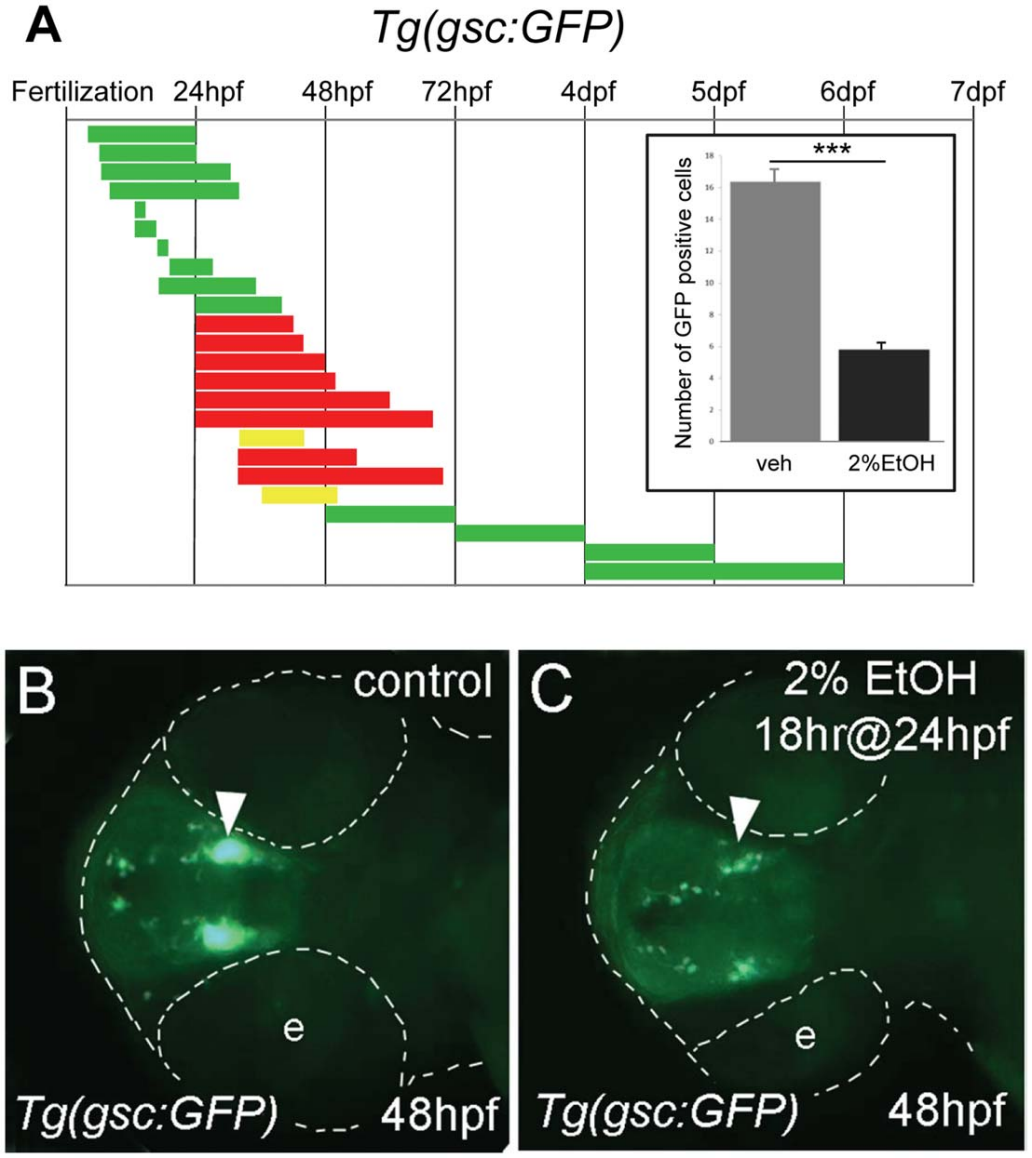
Ethanol exposure reduces the number of *gsc:GFP* expressing neurons. **A**, the duration and embryonic stage of 2% ethanol treatments in *Tg(gsc:GFP)* embryos are illustrated with solid bars. Colors indicate the treatment had no effect (green), slightly reduced (yellow), or reduced (red) GFP expression. **Inset**, the average numbers of GFP positive cells in the preoptic area (POA) of 72 hpf embryos treated with 2% ethanol for 24 hrs at 24 hpf (n = 12) and vehicle control (n = 10). Ethanol treatment resulted in a highly significant reduction of GFP-expressing cells in the POA, ***p<0.001. Error bars represent SEM. **B, C**, ventral views of *Tg(gsc:GFP)* embryos at 48 hpf. **B**, is the control. **C**, treated with 2% ethanol for 18 hrs at 24 hpf. GFP expression in the neuroendocrine preoptic area (arrowheads) was dramatically reduced in the embryos exposed to ethanol. The embryo and eyes (e) are outlined (dashed white line).

The *goosecoid* reporter line, *Tg(−1.8gsc:GFP)ml1*, expressed GFP primarily in two neuronal groups in the neuroendocrine preoptic area (POA). There were also several scattered ectopic cells expressing GFP in olfactory epithelia, epiphysis, tectum, and the eye [49]. The response of *gsc:GFP*-expressing neurons, particularly in the POA, was examined using a wide temporal range of ethanol exposures (Figure 1A). At least ten embryos were examined at multiple developmental stages from each experimental group. The initial experiments utilized a 20 hr exposure of 2% ethanol starting at 4 hours post fertilization (hpf) because this treatment is known to induce apoptosis without grossly perturbing brain morphology or patterning [50], [51]. However, *gsc:GFP* expression was unaffected by these early ethanol treatments.

In contrast, ethanol exposure for 18 or 24 hrs starting at 24 hpf resulted in significantly reduced *gsc:GFP* expression in the neuroendocrine POA (Figure 1B–E). The possibility that ethanol interferes with the differentiation of *gsc:GFP*-expressing neurons was examined using a series of short, developmental time periods covering stages from 19.5 hpf (22 somite) to 25 hpf (prim 8). However, no reduction of *gsc:GFP* expression was observed when examined shortly after exposure or at any stage up to 7 days-post-fertilization (dpf) (Figure S1). Finally, a series of chronic exposures were investigated covering the first six days of embryonic and larval development. These studies demonstrated that *gsc:GFP*-expressing neurons are sensitive to ethanol exposure within a developmental window starting after 24 hpf and ending around 48 hpf. Ethanol treatment after 48 hpf had no effect on *gsc:GFP* expression (Figure S2). Based on our observation of extensive *gsc:GFP* positive tracts by 48 hpf, the developmental window of ethanol sensitivity of *gsc:GFP*-expressing cells is consistent with the predicted period of synaptogenesis of these neurons in the POA.

**Figure 2:**
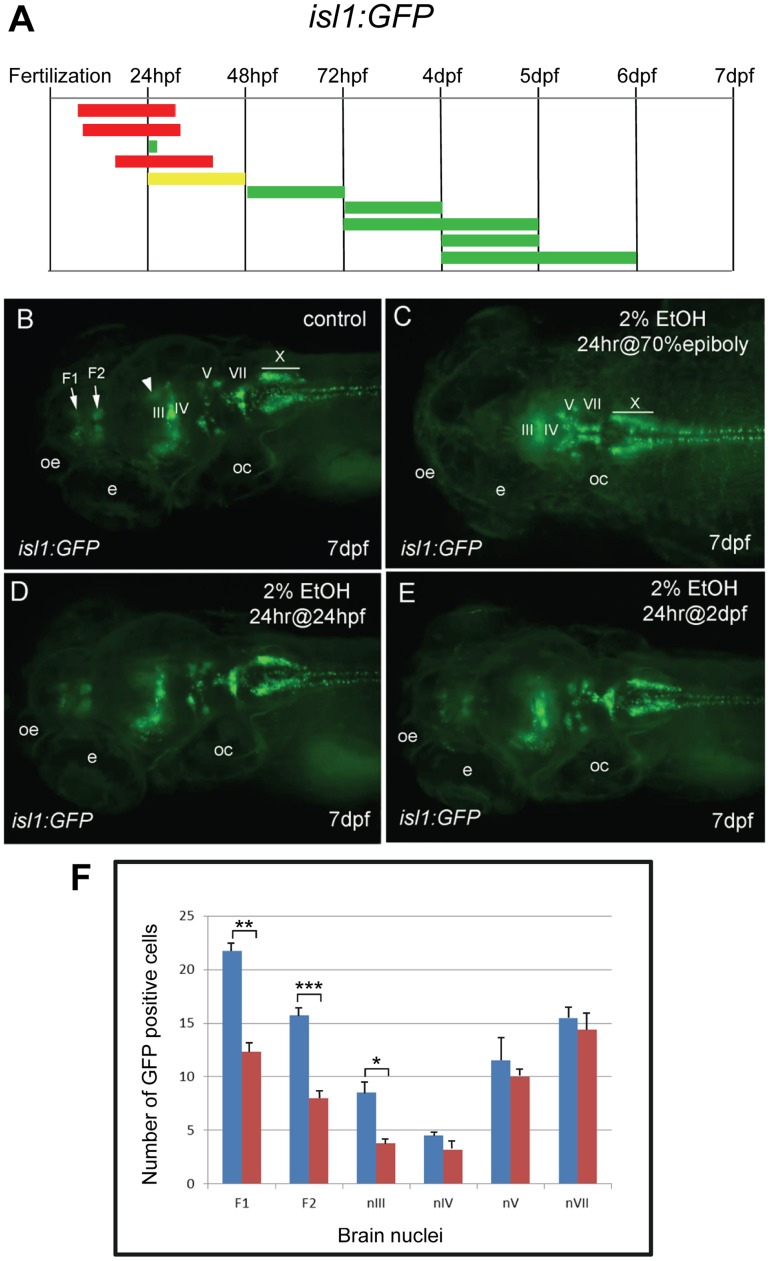
Different groups of neurons are sensitive to ethanol exposure at specific developmental stages. **A**, the duration and embryonic stage of 2% ethanol treatments illustrated with colored bars for *Tg(isl1:GFP)* embryos, as described in Fig. 1. **B−E**, dorsal views of *Tg(isl:GFP)* larva at 7 dpf. **B**, is the control. GFP expression was evident in the forebrain nuclei (F1 and F2 arrows), the mesencephalic cluster (arrowhead) and cranial motor nuclei III, IV, V, VII, and X (labeled). **C**, GFP expression in the forebrain and mesencephalic cluster was absent, and GFP expression in cranial motor nuclei III and IV was reduced (labeled) after 2% ethanol treatment for 24 hrs at the 70% epiboly stage. **D, E**, GFP expression was largely normal after treatment with 2% ethanol for 24 hrs at 24 hpf (D) and for 24 hrs at 48 hpf (E). Eyes (e), otic capsule (oc), olfactory epithelium (oe). **F**, the average numbers of GFP-positive cells in individual brain nuclei of control (n = 10) and ethanol-treated (n = 10) embryos. A highly significant reduction in GFP-expressing cells in the two forebrain nuclei, F1 (*p* = 0.001) and F2 (*p*<0.001), and cranial motor nucleus III (*p*<0.05) was observed. In contrast, a slight reduction in GFP cells in motor nuclei IV, V and VII was not significant. Error bars represent SEM. ***p<0.001, **p<0.01, *p<0.05.

To determine the effect of ethanol exposure on other neurons, we used *isl1:GFP* and *pax2a:GFP* transgenic lines [52], [53]. *Tg(isl1:GFP)rw0* embryos express GFP in select forebrain nuclei, a mesencephalic cluster, in cranial motor neurons III, IV, V, VII and X, in facial, glossopharyngeal and vagal sensory ganglia, and in Rohan-Beard primary sensory neurons and motor neurons of the spinal cord [52]. A series of chronic exposures covering the first six days of development was used to identify ethanol-sensitive periods (Figure 2A). Ethanol exposure for 24 hrs starting at 70% epiboly abolished *isl1:GFP* expression in the two forebrain nuclei and the mesencephalic cluster, and reduced expression in cranial motor nuclei III and IV (Figure 2B–F). In contrast, ethanol treatment for 24 hrs at 24 hpf only resulted in a slight and variable reduction of *isl1:GFP* expression in the forebrain nuclei, and treatment later than 48 hrs had no effect on *isl1:GFP* expression (Figure S3). This indicates that *isl1:GFP*-expressing forebrain neurons are sensitive to ethanol during an earlier developmental period than the *gsc:GFP*-expressing POA neurons.

**Figure 3:**
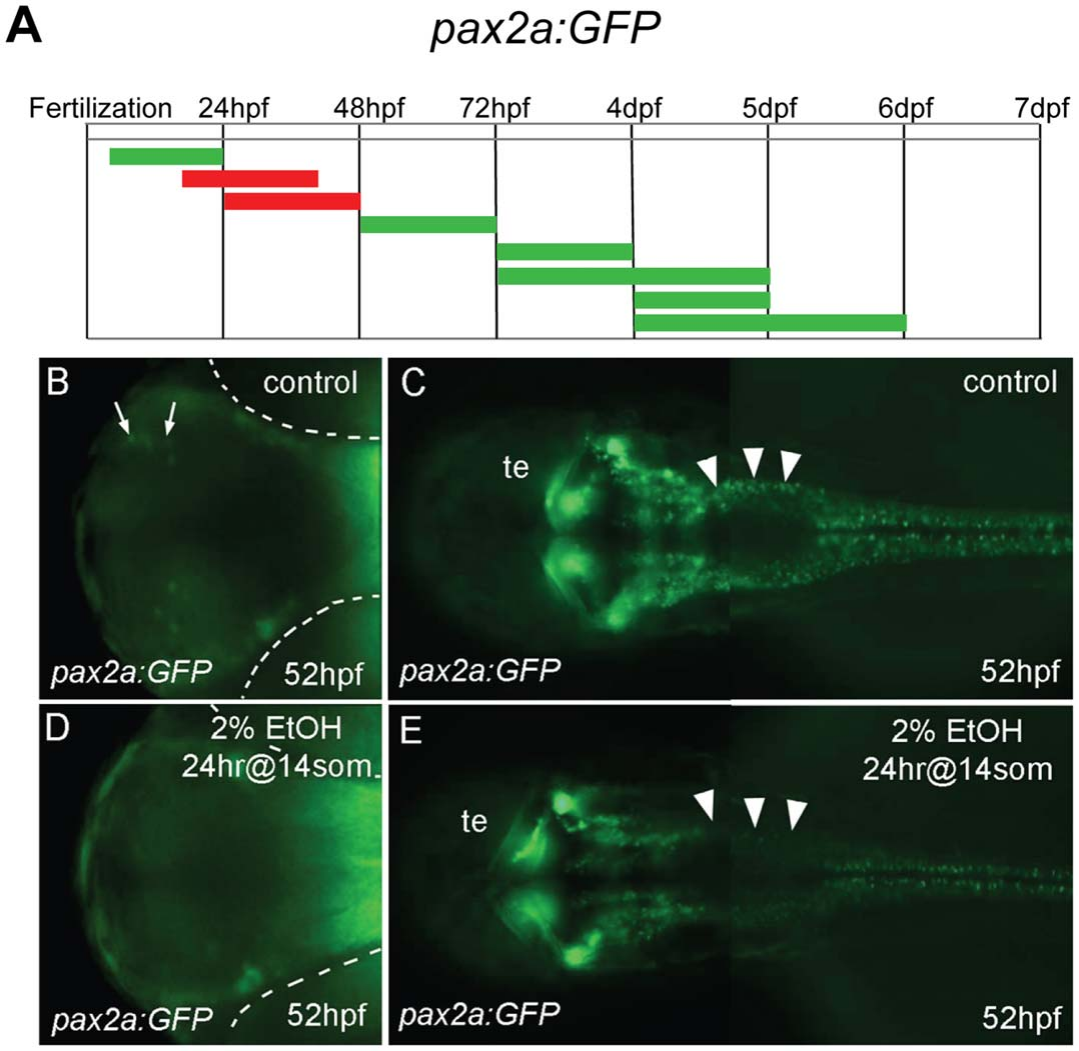
Forebrain and hindbrain *pax2a:GFP* positive cells are sensitive to ethanol exposure. **A**, the duration and embryonic stage of 2% ethanol treatments illustrated with colored bars for *Tg(pax2a:GFP)* embryos, as described in Fig. 1. **B−E**, ventral views (B, D) and dorsal views (C, E) of *Tg(pax2a:GFP)* embryos at 52 hpf. **B, C**, are controls. **D, E**, were treated with 2% ethanol for 24 hrs at the 14 somite stage. **B, D**, a small, distinct group of neurons in the forebrain (arrows in B) were missing after ethanol exposure (D). The location of the eyes is outlined (dashed white lines). **C, E**, neurons around the midbrain-hindbrain region were mostly unaffected, hindbrain neurons were drastically reduced (arrowheads), and spinal cord neurons appeared slightly reduced. Tectum (te).

*Tg(pax2a:GFP)e1* (pGFP5.3) is expressed in the mid-hindbrain border, rhombomeres 3 and 5, one telencephalic area and three diencephalic areas, as well as in the VIIIth cranial nerve ganglion, the hindbrain and spinal cord interneurons [53]. Early ethanol exposure, from dome stage to 24 hpf, did not appear to affect *pax2a:GFP* expression, whereas treatment for 24 hrs starting at the 14 somite stage or at 24 hpf resulted in an apparent reduction of *pax2a:GFP*-expressing neurons in the hindbrain. In addition, a small group of *pax2a:GFP* neurons were eliminated in the forebrain (Figure 3A–E). Interestingly, *isl1:GFP*-expressing neurons in the hindbrain were unaffected by ethanol treatment.

*Tg(elavl3:EGFP)knu3* (also known as *HuC:EGFP*), which expresses GFP in most post-mitotic neurons, was used to observe the effect of ethanol treatments on overall neuronal development [54]. *Tg(elavl3:EGFP)knu3* embryos were exposed to a range of ethanol concentrations, from 0.5% to 2.0%, for various durations at several developmental stages. In all cases, no general effects on post-mitotic neurons were observed, suggesting that these ethanol exposures are disrupting development of highly specific subsets of neurons (Figure S4, S5).

### Neurons are Sensitized to Ethanol Exposure in med12 Mutant Embryos

**Figure 4:**
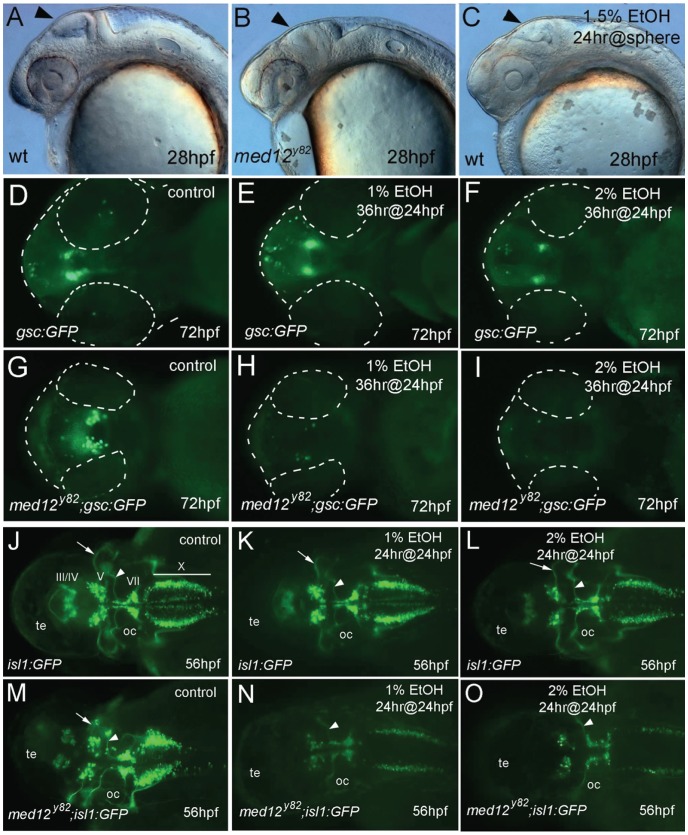
Neurons are sensitized to ethanol in *med12* mutant embryos. A−C , 28 hpf embryos. **A**, wild-type (wt), **B**, *med12^y82^* mutant, and **C**, wt embryo treated at sphere stage with 1.5% ethanol for 24 hrs. Note the similarly retarded brain development in the *med12^y82^* and ethanol treated embryos (arrows). **D−I**, ventral views at 72 hpf of *Tg(gsc:GFP)* embryos (D, E, F) and *med12^y82^*; *Tg(gsc:GFP)* embryos (G, H, I). **D, G**, control embryos. **E, H**, treated with 1% ethanol for 36 hrs at 24 hpf. **F, I**, treated with 2% ethanol for 36 hrs at 24 hpf. Embryos and eyes are outlined (dashed white lines). **J−O**, *Tg(isl1:GFP)* embryos (J, K, L) and *med12^y82^*; *Tg(isl1:GFP)* embryos (M, N, O) at 56 hpf, dorsal views. **J, M**, control embryos. **K, N**, treated with 1% ethanol for 24 hrs at 24 hpf. **L, O**, treated with 2% ethanol for 24 hrs at 24 hpf. GFP-expressing cranial motor neurons (labeled) diminished in *med12^y82^*; *Tg(isl1:GFP)* embryos, especially in nuclei III and IV. A reduction of nucleus V neurons in *med12^y82^*; *Tg(gsc:GFP)* embryos treated with ethanol is illustrated by the elimination of the prominent nV axons (arrows). In contrast, the prominent nVII axons remained intact (arrowheads). Otic capsule (oc), tectum (te).

As a first step toward understanding the influence of genetic factors on neuronal sensitivity to ethanol, we focused on the zebrafish mutant, *med12^y82^*. The *med12^y82^* mutation is a point mutation in the mediator complex gene *med12* that creates a premature stop codon, inactivating this protein [48]. Mutations in the human *MED12* gene result in a variable mental retardation syndrome with behavioral disturbances [55], [56]. In zebrafish, *med12^y82/y82^* embryos have several features, particularly brain anomalies that appear similar to embryos that have been treated with ethanol (Figure 4A–C). Therefore, we examined ethanol sensitivity in *med12^y82^* mutants that had been crossed into the four transgenic GFP reporter lines.

Homozygous *med12^y82/y82^* mutations resulted in severe deficits in several neuronal populations. Ethanol treatment, for the most part, resulted in further neuronal defects in *med12* mutant embryos. Interestingly, ethanol exposure at concentrations below those that effect normal embryos resulted in significant enhancement of deficits in *med12^y82/y82^* embryos. In contrast, the ethanol response in heterozygous *med12^y82/+^* embryos was indistinguishable from that in wild-type embryos. In *med12^y82/y82^*; *Tg(elavl3:EGFP)knu3* embryos, overall numbers of *elavl3:GFP*-expressing post-mitotic neurons were reduced compared to their wild-type and heterozygous siblings. Ethanol treatment further reduced *elavl3:GFP* expression in *med12^y82/y82^* embryos, but not in wild-type and heterozygous siblings. However, no obvious region-specific reduction in *elavl3:GFP* expression was observed in any of the groups (Figure S5A–F). The level of *elavl3:GFP* expression in 48 hpf embryos was further evaluated by real-time qRT-PCR. The expression of GFP mRNA was normalized to *β-actin* mRNA levels and relative expression was determined for 1) *Tg(elavl3:EGFP)* embryos treated with 2% ethanol for 24 hrs at 24 hpf, 2) *Tg(elavl3:EGFP)* control embryos, 3) *med12^y82/y82^*; *Tg(elavl3:EGFP)* embryos treated with 2% ethanol for 24 hrs at 24 hpf, and 4) *med12^y82/y82^*; *Tg(elavl3:EGFP)* control embryos. The relative expression of *elavl3:GFP* mRNA was not significantly different among the four groups. However, there appears to be a trend toward higher relative levels of *elavl3:GFP* mRNA, normalized to *β-actin* mRNA, in *med12* mutant and ethanol-treated embryos (Figure S5G). These results suggest that there is not a specific loss of post-mitotic neurons due to the *med12* mutation or to ethanol treatment.

Embryos from *med12^y82^;Tg(−1.8gsc:GFP)* incrosses were treated with 1% or 2% ethanol for 36 hrs at 24 hpf. The expression of GFP in the POA of *Tg(−1.8gsc:GFP)* embryos was reduced by exposure to 2% but unaffected by 1% ethanol. In contrast, exposure of *med12^y82/y82^;Tg(−1.8gsc:GFP)* embryos to 1% ethanol resulted in the elimination of *gsc:GFP* expression in the POA, while untreated *med12^y82/y82^;Tg(−1.8gsc:GFP)* embryos had variably reduced and disorganized *gsc:GFP*-expressing neurons (Figure 4D–I). These results demonstrate sensitization to ethanol exposure in embryos with a *med12* mutant genetic background.

GFP expression in *med12^y82/y82^;Tg(isl1:GFP)* embryos was absent in the forebrain, highly reduced in cranial motorneuron nuclei III and IV, and displayed moderately reduced expression overall. After exposure to 1% ethanol, which had no effect on *isl:GFP* expression in wild-type or heterozygous embryos, *isl:GFP* expression in *med12^y82/y82^* embryos was further reduced. When treated with 2% ethanol, *isl:GFP* expression in *med12^y82/y82^* embryos was nearly absent (Figure 4J–O).

The early GFP expression in *med12^y82/y82^;Tg(pax2a:GFP)* embryos was similar to wild-type *Tg(pax2a:GFP)* embryos at 24 hpf (Figure S6). However, by 60 hpf, hindbrain and spinal cord neuronal expression was greatly reduced in *med12^y82/y82^;Tg(pax2a:GFP)* embryos; 2% ethanol treatment resulted in the near elimination of these neurons, while midbrain-hindbrain border and rhombomeric pattern expression remained fairly normal (Figure S7). These results indicate that while patterning is largely unaffected by *med12* mutation or ethanol exposure, differentiating *pax2a:GFP* neurons are highly sensitive to these perturbations.

**Figure 5:**
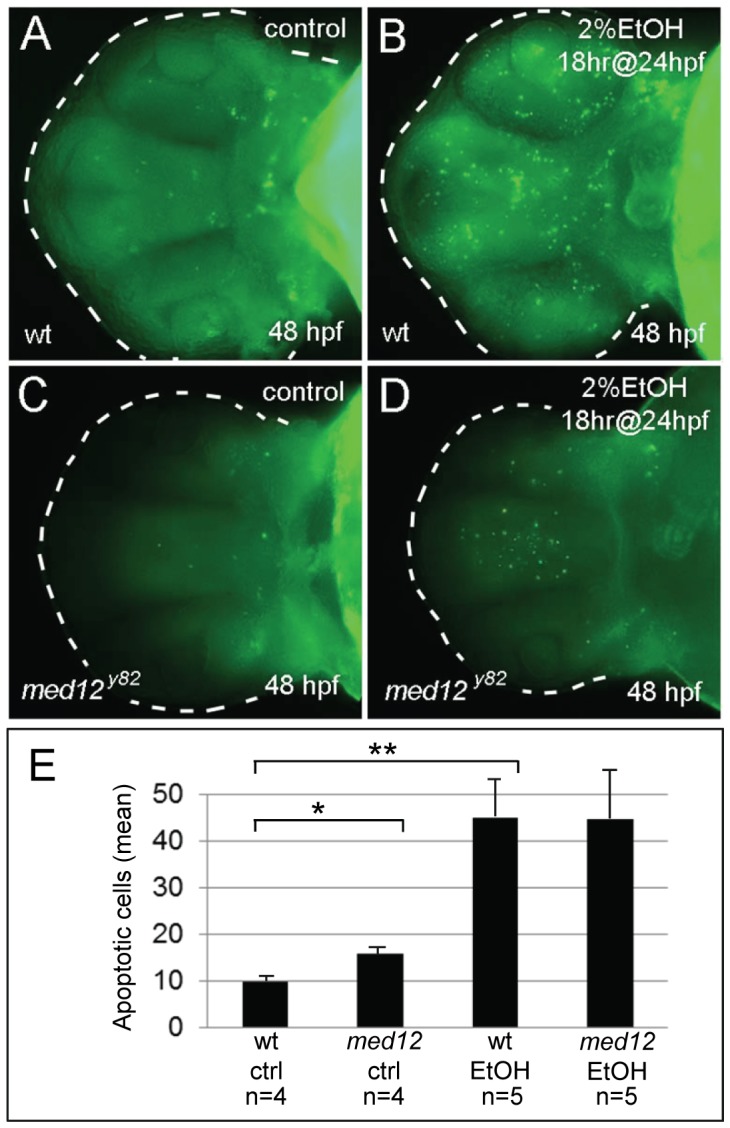
Ethanol exposure causes similar levels of neuronal cell death in wild-type and *med12* mutant embryos. **A−D**, acridine orange stained embryos at 48 hpf, ventral views. **A**, untreated wild-type (wt) embryo. **B**, wt embryo treated with 2% ethanol for 18 hrs at 24 hpf. **C**, untreated *med12^y82^* embryo. *Med12^y82^* and wt embryos showed similar low levels of apoptosis. **D**, *med12^y82^* embryo treated with 2% ethanol for 18 hrs at 24 hpf. *Med12^y82^* and wt embryos treated with 2% ethanol showed similar increases in acridine orange staining. **E**, the average numbers of apoptotic cells in the brains of *med12^y82^* and control embryos treated with ethanol as in panels A−D. There were significantly more apoptotic cells in the brains of *med12^y82^* embryos compared to wild-type embryos (*p*<0.05), however, following EtOH exposure, the numbers of apoptotic cells in the brains of both *med12^y82^* and wild-type embryos greatly increased. The number of apoptotic cells in the brains of *med12* mutant embryos increased 2.8-fold compared to their untreated *med12* mutant siblings (*p* = 0.073), while apoptotic cells increased over 5-fold in ethanol-treated wild-type embryos compared to their untreated siblings (*p*<0.005). Error bars represent SEM. **p<0.01, *p<0.05.

In order to investigate cell death as a mechanism by which ethanol potentiates the neuronal deficits of *med12* mutants, wild-type and *med12^y82/y82^* embryos were treated with 2% ethanol for 18 hours at 24 hpf, and then stained with acridine orange at 48 hpf to reveal apoptotic and necrotic cells. In addition, embryos were fixed and sectioned, and then stained for apoptotic cells using a TMR based TUNEL assay for quantitative analyses. Untreated wild-type and *med12^y82/y82^* embryos showed relatively low-levels of acridine orange staining [48], [57]. In contrast, ethanol treatment induced high-levels of acridine orange staining in both wild-type and *med12^y82/y82^* embryos. Quantitative analysis of TUNEL stained sections confirmed the acridine orange results. Untreated wild-type and *med12^y82/y82^* embryos showed relatively low levels of apoptosis, however, *med12^y82/y82^* embryos had relatively more apoptotic cells than their wild-type or heterozygous siblings (*p*<0.05). Nevertheless, it is unlikely that this modest increase in apoptosis could account for the increased sensitivity to ethanol observed in *med12* mutant embryos considering the dramatic increase in apoptosis observed following ethanol exposure (Figure 5A–E). Ethanol exposure for 24 hrs starting at 24 hpf resulted in a 5.7 fold increase in apoptotic cells in wild-type embryos (*p*<0.005) and a 2.8 fold increase in med12 embryos (*p* = 0.073).

### Transgenic oxtl:GFP Reporter Lines Reveal Ethanol Induction of Oxytocin in the Hindbrain

**Figure 6:**
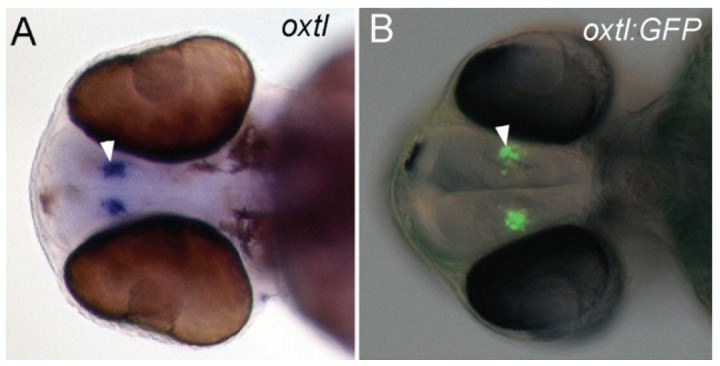
Expression of GFP in *Tg(oxtl:GFP)* embryos corresponds exactly with the expression of endogenous *oxtl* mRNA. **A,B**, ventral view of 48 hpf embryos. **A**, whole-mount *in situ* hybridization for *oxtl* mRNA in a 48 hpf embryo. **B**, GFP expression visualized with epi-fluorescence in a 48 hpf *Tg(oxtl:GFP)* embryo. Epi-fluorescent and differential interference contrast (DIC) images are overlaid. The *oxtl* expressing cells are indicated with arrowheads.

**Figure 7:**
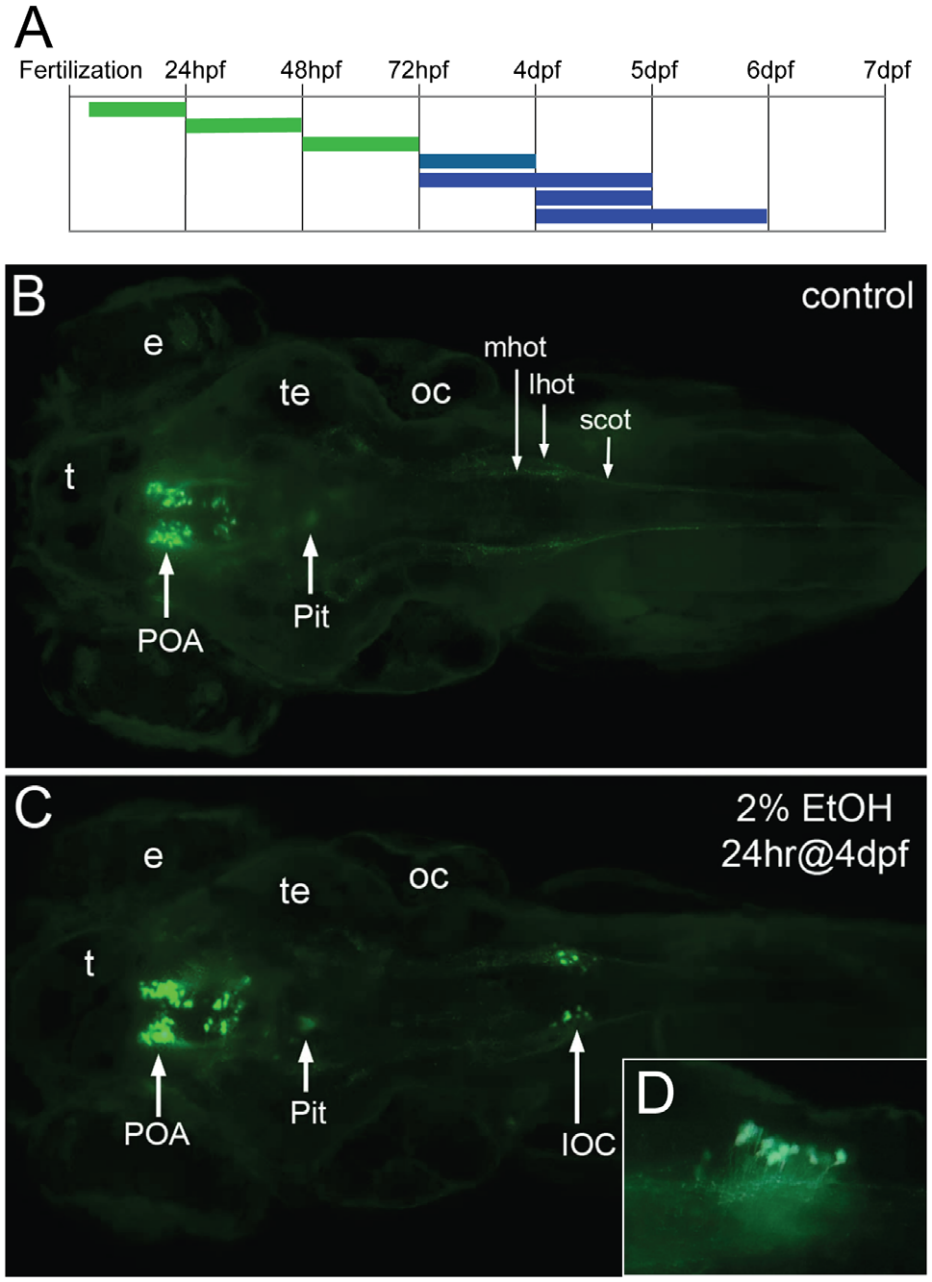
Expression of the *oxtl:GFP* transgene is induced in the hindbrain by exposure to ethanol. **A**, the duration and embryonic stage of 2% ethanol treatments are illustrated with solid bars. *Oxtl:GFP* expression in the neuroendocrine preoptic area was unaffected with all treatments (green), while *oxtl:GFP* was induced in the hindbrain by later treatments (blue). **B, C**, *Tg(oxtl:GFP)* larva shown at 6 dpf, dorsal views. **B**, control embryo, **C**, treated with 2% ethanol for 24 hrs at 4 dpf. A small group of large GFP-expressing neurons appears in the hindbrain of the ethanol-treated larva. We call these ethanol-induced GFP-expressing neurons, induced oxytocin cells (IOC). **D, inset**, a higher magnification view of the IOCs shows processes that appear to enter the hindbrain oxytocinergic tracks, lateral view. Eyes (e), lateral hindbrain oxytocinergic tract (lhot), medial hindbrain oxytocinergic tract (mhot), otic capsule (oc), pituitary (Pit), preoptic area (POA), spinal cord oxytocinergic tract (scot), tectum (te), telencephalon (t).

In order to test the hypothesis that the developing oxytocinergic system would be particularly sensitive to alcohol exposure, we generated transgenic lines that express GFP from the *oxtl* promoter. In *Tg(oxtl:GFP)* embryos, GFP expression corresponds exactly with the expression of endogenous *oxtl* mRNA as determined by ISH at 48 hpf (Figure 6). A more detailed neuroanatomical analysis of the oxytocinergic system based on the *Tg(oxtl:GFP)* lines will be published elsewhere, though here we highlight the more striking features of these fish. As expected, oxytocinergic cell bodies in the POA prominently express GFP with tracts to the pituitary. A surprising feature of these fish is the presence of extensive tracts and projections throughout the mid- and hindbrain, with tracts continuing down the spinal cord (Figure 7B). While “central” projection patterns are well-known features of the oxytocinergic system, our use of zebrafish allows us to finally visualize and appreciate the full extent of oxytocinergic projections in a living organism.

A series of ethanol exposures was used to investigate developmental windows of alcohol sensitivity in the oxytocinergic system (Figure 7A). Ethanol exposure in *Tg(oxtl:GFP)* embryos did not alter cell numbers or projection patterns nor projection density of GFP-expressing neurons in the POA. Surprisingly, however, a small group of large GFP-expressing neurons appeared in the posterior hindbrain of *Tg(oxtl:GFP)* larva treated with 2% ethanol for 24 hrs at 4 dpf (Figure 7B–D). We call these ethanol-induced *oxtl:GFP*-expressing neurons, Induced *Oxtl* Cells (IOC). IOCs appear in the posterior region of the hindbrain, dorsal and medial to where at least two hindbrain oxytocinergic tracks converge to form a single tract in the spinal cord (compare 7B and 7C). The position of the IOCs suggest they are in the area postrema, or are, perhaps, part of the posterior most nucleus of the solitary tract, and thus receive afferent visceral innervations. Processes appear to exit the IOCs and join the hindbrain oxytocinergic tracks (Figure 7D).

**Figure 8:**
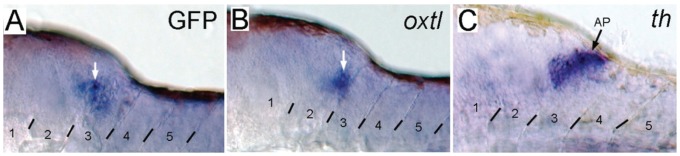
Expression of *oxtl* mRNA is induced in the hindbrain by exposure to ethanol. Whole mount *in situ* hybridization was used to detect: **A**, *GFP*; **B**, *oxtl*; and **C**, *tyrosine hydroxylase* (*th*) mRNAs in *Tg(oxtl:GFP)* larva treated with ethanol for 24 hrs at 4 dpf, and then fixed at 6 dpf. *th* labels dopaminergic and noradrenergic neurons located in the area postrema. Examples of positive staining are indicated by arrows.

To confirm that these GFP-expressing IOC’s also express endogenous *oxtl*, *in situ* hybridization (ISH) was used to detect *GFP*, *oxtl* and *tyrosine hydroxylase* (*th*) mRNAs in *Tg(oxtl:GFP)* larva treated with ethanol for 24 hrs at 4 dpf, and then fixed at 6 dpf (Figure 8A–C). The presence of *GFP* and *oxtl* mRNA in the posterior hindbrain of ethanol-treated larva shows that induction of GFP expression coincides with the induction of endogenous *oxtl* mRNA. *th* expression in the posterior hindbrain marks the area postrema. The induced GFP/*oxtl* cells appear to be slightly inferior and medial to the *th* positive cells. These results demonstrate that *oxtl* is induced in the hindbrain by ethanol exposure.

## Discussion

### Alcohol Exposure Results in Complex Changes in Brain Development

It is likely that a number of mechanisms are responsible for the deleterious effects of alcohol. Although several potential mechanisms have been demonstrated in culture systems, the importance and relative contribution of the various mechanisms to lasting damage in the brain remains unclear [16], [19], [25]. Substantial efforts have been devoted to documenting changes in the brains of model organisms that have been exposed to ethanol and these studies have significantly contributed to our understanding of ethanol neurotoxicity [16]–[18], [20]–[24]. However, given the difficulty of controlling alcohol dose and timing and the requirement for static post-mortem analyses, these studies are unable to capture the full range of effects of ethanol concentration, duration of exposure, and embryonic stage of exposure on brain development. Furthermore, the relative importance of various mechanisms of ethanol neurotoxicity may change depending on cell type, dose, duration, and developmental stage of exposure.

By utilizing transgenic zebrafish to monitor brain development, we demonstrate that a wide range of exposures can be rapidly evaluated to identify sensitive subsets of neurons. Several treatment regimens have been used in zebrafish that result in morphologic abnormalities that closely model FAS [58]. Because we are interested in modeling the more subtle neurodevelopmental deficits of FASD, we used ethanol doses that are below those that generate FAS like phenotypes. An advantage of using zebrafish embryos and larva is that dosing is easy and accurate; therefore, it is possible to obtain sensitivity data for any given group of neurons over a wide parameter space consisting of ethanol dose, duration and developmental stage of exposure. Given the large and growing collection of enhancer-specific transgenic lines, it should be feasible to generate a comprehensive map of neuronal sensitivity to ethanol exposure [35]–[41]. Such a map would greatly advance our understanding of the complex effects that alcohol exposure has on brain development. Furthermore, the ability to perform forward genetic screens in zebrafish and the large collection of mutant lines already available will enable investigation of the interaction of ethanol exposure with underlying genetic mutations.

### Neurons are Sensitized to Ethanol in *med12* Mutant Embryos

Mutations in the human *MED12* gene result in X-linked mental retardation disorders, including Opitz-Karreggia syndrome and Lujan syndrome, and these mutations are also associated with schizophrenia [13]–[15], [59], [60]. Some overlapping clinical manifestations of these syndromes, including agenesis of the corpus callosum, behavioral disturbances and cognitive deficits, are reminiscent of FASD [15], [27], [28]. Our results demonstrate a heightened sensitivity to ethanol-induced deficits in select groups of neurons in *med12* mutant embryos (Figure 3).

The sensitization to ethanol exposure observed in *med12* mutants suggests a synergistic interaction, potentially through an increase in cell death. Several mechanisms of ethanol’s effect on brain development have been proposed to act by inducing apoptosis [21], [61], [62]. Ethanol-induced increases in apoptosis can be observed by acridine orange staining. However, in wild-type embryos, acridine orange staining is not restricted to specific groups of cells. Rather, apoptotic cells appear to be randomly distributed throughout the neuraxis, an observation that argues against increased apoptosis as a mechanism for the ethanol-induced reduction of specific neuronal populations. Nevertheless, ethanol exposure in *med12* mutant embryos could sensitize certain neurons to apoptosis-triggering events. In *med12* mutant embryos, acridine orange and TUNEL staining shows a modest increase in apoptosis in comparison to wild-type embryos [48], [57]. In contrast, the number of apoptotic cells increase dramatically in both *med12* mutant and wild-type embryos following ethanol exposure. In addition, the levels of apoptosis are similar between ethanol treated wild-type and *med12* mutant embryos suggesting that the increased sensitivity of subsets of neuronal cells to ethanol exposure in *med12* mutant embryos is unlikely to be explained by increased apoptosis.

### Novel *Oxytocin* Expression in the Hindbrain is Induced by Ethanol Exposure

In order to utilize zebrafish for the analysis of the effect of embryonic alcohol exposure on the oxytocinergic system, we generated *oxtl:GFP* transgenic embryos. The oxytocinergic system was chosen because this system, across a spectrum of vertebrate animals, is closely associated with social behaviors, the disruption of which is one aspect of FASD [29]–[33]. Significantly, zebrafish display defects in social behavior following embryonic ethanol exposure [43], [44], [46], [63]. Thus, we anticipated that this type of exposure would lead to deficits in the oxytocinergic system.

Surprisingly, ethanol exposure induced *oxtl:GFP* expression in the hindbrain, while not affecting the oxytocinergic system of the POA in *oxtl:GFP* transgenic larva. The induction of oxytocinergic cells in the hindbrain has not previously been observed in any vertebrate organism. However, ethanol-stimulated induction of hindbrain oxytocinergic neurons in mammals, including humans, would not be an unreasonable phenomenon to expect. The lack of previously observed oxytocin-expressing cells in the mammalian hindbrain may simply be due to the relative difficulty of visualizing specific neuronal phenotypes in the mammalian brain. We were able to observe these *oxtl*-expressing neurons because the entire nervous system is visible in the zebrafish, a feature of this model that facilitates unexpected discoveries.

The induction of novel neurotransmitter phenotypes as a consequence of fetal alcohol exposure has not been reported previously. In nearly all cases, whether in cell culture, tissue culture or intact animals, ethanol exposure results in decreased proliferation, neuronal dysfunction or cell death [16], [19], [25]. In contrast, we observe robust induction of *oxtl* expression in the hindbrain of the zebrafish following ethanol exposure.

These observations invite speculation on the potential function of IOCs in the hindbrain and the consequences of their expression in the larva. IOCs arise in the posterior hindbrain, most likely the area postrema. In mammals, the area postrema detects toxins in the blood and is excited by visceral afferent impulses (sympathetic and vagal) arising from the gastrointestinal tract and other peripheral trigger areas [64], [65]. This brain region also contains numerous dopaminergic and serotonergic neurons, which could indicate an interaction between IOCs and these systems, as interactions among the oxytocinergic, dopaminergic and serotonergic systems in the forebrain have been proposed to mediate a host of behaviors [43], [66]–[71]. Furthermore, the area postrema contributes to behavioral responses to blood-borne chemicals, such as taste aversion learning in rats [72]–[74]. Oxytocin has strong effects on behavior through mediating positive feelings of social interactions [29]–[33], [75]. Interestingly, addictive “party” drugs and alcohol increase oxytocin levels, and oxytocin treatment reduces drug self-administration behavior and improves withdrawal symptoms [76], [77]. Based on our observation that alcohol exposure induces *oxtl* expression in the area postrema, we hypothesize that oxytocin release from area postrema neurons mediates drug-seeking and addictive behavior. Furthermore, we hypothesize that embryonic and/or fetal exposure to ethanol sensitizes IOCs such that they potentiate positive feelings upon re-exposure in later life, and this may partially explain the high incidence of alcoholism and other addictive behaviors observed in FASD [5], [6], [8], [9], [78]–[83].

In conclusion, we have discovered a new phenomenon resulting from ethanol exposure, namely, the induction of *oxtl*-expressing neurons in the hindbrain. This discovery highlights an under-appreciated advantage of the zebrafish model: The effects of ethanol exposure on brain development, in both wild-type and mutant embryos, can be visualized in the entire embryo at a cellular level. Thus, events occurring at unexpected locations can be discovered in the process of studying seemingly unrelated phenomena. The discovery of induced *oxtl* expression in the hindbrain may have profound implications for understanding alcoholism and other addictive disorders.

## Materials and Methods

### Animals

Zebrafish (*Danio rerio*) were raised, maintained and crossed as described [84]. Development of embryos was at 28°C, and staging was determined by both hpf and morphological characteristics [85]. All procedures were in accordance with NIH guidelines on the care and use of animals and were approved by the Georgetown University Institutional Animal Care and Use Committee, Protocol 11-008.

### EtOH Treatments

Ethanol solutions were prepared by diluting 95% ethanol (Molecular Biology Grade, Sigma-Aldrich) in fish water (0.3 gm/L sea salt, Marine Biosystems) with or without 30 mg/L phenylthiourea (Sigma, P7629). Embryos were collected from pair-wise or group mating, debris and unfertilized eggs were removed, and embryos were placed in fish water. Fish water was removed by pipetting or pouring, and the embryos or larva were rinsed once in ethanol solution, and then fresh ethanol solution was added. The number of embryos/larva and the volume of ethanol solution were equivalent for all treatment groups within any given experiment, but varied from experiment to experiment. All culture dishes were sealed with parafilm for the duration of the ethanol treatments. Following treatment, the ethanol solutions were removed, the dish was rinsed once with fish water and the embryos were raised in fish water until observation.

### Whole-mount *in situ* Hybridization

The whole-mount *in situ* hybridization procedures were performed as previously detailed [86]. *Oxtl* (*ist*) antisense riboprobe was synthesized using SP6 RNA polymerase from EcoRI linearized template cDNA [34].

*tyrosine hydroxylase* (*th*) was cloned by reverse transcriptase polymerase chain reaction (RT-PCR) using the following primers: th-RT1, 5′-CAACACATTCAGGGCATCTG-3′; th-F1, 5′-CAGCTCCACATCTTCCACAAA-3′; and th-R1, 5′-ACAGAAAACGGTCGCTTGAT -3′. Total RNA was isolated from 3 dpf embryos using TRIzol Reagent (Invitrogen, 15596-026). *th* RNA was reverse transcribed using the *th*-RT1 primer with SuperScript III First-Strand Synthesis Supermix (Invitrogen, 18080-400). *th* cDNA was amplified using 2x PCR Master Mix (Fermentas, #K0171) with primers *th*-F1 and *th*-R1. The PCR product was digested with NotI and HindIII, releasing a 1,138 bp internal *th* fragment, which was gel purified and cloned into pBluescript II (Agilent). *th* antisense riboprobe was synthesized from NotI linearized template cDNA using T7 RNA polymerase following the protocol in the DIG RNA Labeling Kit (Roche, Indianapolis, IN).

GFP cDNA template was generated by amplification using 2x PCR Master Mix (Fermentas, #K0171) with primers: *gfp*F1, 5′-GACGGCGACGTAAACGGCCA-3′; and *gfp*R1-T7, 5′-TAATACGACTCACTATAGGGCTTGCTCAGGGCGGACTGGGT-3′. The T7 sequence is underlined. *EGFP* antisense riboprobe was synthesized from PCR amplified GFP template using T7 RNA polymerase following the protocol in the DIG RNA Labeling Kit (Roche, Indianapolis, IN).

### Isolation and Characterization of Zebrafish *oxtl* Genomic Clones

Genomic clones representing zebrafish *oxtl* were isolated by screening an arrayed zebrafish P1-derived Artificial Chromosome (PAC) library obtained from the Resource Center, German Human Genome Project (RZPD library number, 706), and hybridized with a zebrafish [P^32^]-labeled *oxtl* cDNA probe. Three clones hybridized strongly with the probe and were therefore obtained from the RZPD. These clones were characterized by restriction endonuclease mapping and Southern hybridization analysis, which shows that they overlap the *oxtl* transcribed region. We chose the largest *oxtl* clone (IT2.4) for subcloning and limited sequencing. The sequence from this PAC clone was used to design PCR primers to amplify potential regulatory regions.

### Generation of oxtl:EGFP Transgenic Lines

A 1.7 kb region of 5′ *oxtl* sequence was amplified from the PAC clone IT2.4 using the following primers with restriction sites added at their 5′ ends to facilitate cloning:

Xho-ISTG-F1 5′-GAGACTCGAGCACAGTTGATTCATGTTGAGCA.

ISTG-R1-BamHI 5′-CGGGATCCACTGTGGAGGAAGAGACGTACA.

The amplified region contains 580 bp upstream of the transcriptional start site, the first exon (non-coding) of *oxtl*, the first intron, and a single bp of the second exon two bp upstream of the translational start site. This region was cloned into the Tol2 vector pT2AL200R150G at unique XhoI to BamHI sites, replacing the Xenopus EF1a enhancer-promoter and rabbit b-globin intron, with the *oxtl* sequence [87], [88]. The resulting plasmid, pT2-1.7*oxtl:GFP*, was confirmed by sequencing. Capped Tol2 transposase mRNA was synthesized from the pCS2FA-transposase plasmid as described [89]. About 450 embryos were injected with supercoiled pT2-1.*7oxtl:GFP* plasmid plus Tol2 transposase RNA as described [90]. Approximately 70% of the 205 surviving embryos expressed GFP in an *oxtl* pattern [34].

Seven independent lines that expressed GFP in an *oxtl* pattern were recovered from 32 outcrossed fish. Three lines with the brightest expression were maintained and used for ethanol exposure experiments.

### Acridine Orange, TUNEL Staining and Microscopy

Acridine orange staining was done as previously described [91]. Briefly, embryos were manually dechorionated, anesthetized in 0.016% tricaine (Sigma), and then incubated in 50 ng/ml acridine orange (Invitrogen, A1301) for 1hr. Following several washes in fish water, the embryos were mounted in 3% methyl cellulose and imaged. For TUNEL staining, 48 hpf embryos were fixed in 4% paraformaldehyde, embedded in agarose, and then sectioned on a vibratome. Apoptotic cells were visualized using an In Situ Cell Death Detection Kit, TMR red (Roche Applied Science) as per the manufacturer’s instructions. Transgenic embryos were anesthetized then mounted in 3% methyl cellulose or 1% low-melt agarose. Images were acquired using a Zeiss Axioplan2 microscope fitted with an AxioCam camera using AxioVision software.

### Statistical Analysis

Statistical analyses were performed using SPSS for Windows (version 20.0.1). Quantitative analyses for GFP-positive cell numbers in specific brain nuclei were performed using unpaired (two-tailed) student’s t test.

## Supplemental Methods

### RNA Isolation and Real-time Quantitative RT-PCR

RNA was isolated from groups of ten embryos using TRIzol Reagent (Invitrogen). One ug RNA was then converted to cDNA using the High Capacity RNA-to-DNA kit (Applied Biosystems). Reactions containing Fast SYBR Green master mix (Applied Biosystems), cDNA and primers were amplified using an Applied Biosystems 7900HT Fast Real-Time System. The following primers were used: GFP_F1 5′-AGCCGCTACCCCGACCACAT;

GFP_R1 5′-TGCGCTCCTGGACGTAGCCT; bactin2F1 5′-CGAGCTGTCTTCCCATCCA; bactin2F1 5′-CGAGCTGTCTTCCCATCCA.

Relative Quantitation was analyzed in RQ Manager 1.2 software using the Comparative C_T_ method.

## Supporting Information

Figure S1 **Ethanol exposure during the predicted time period of initial differentiation of** ***gsc*** **-expressing cells had no effect on** ***gsc:GFP*** **expression. A–D**, *Tg(gsc:GFP)* larval at 6 dpf, dorsal views. **A**, is control. **B**, treated with 2% ethanol from 11 to 17 somites stage. **C**, treated with 2% ethanol from 19 to 25 somites stage. **D**, treated with 2% ethanol from 22 somites to prim8 stage.
(TIF)

Figure S2 **Ethanol exposure later than 48 hpf had no effect on** ***gsc:GFP*** **expression. A–F**, *Tg(gsc:GFP)* larval at 7 dpf, dorsal views. **A**, is control. **B**, treated with 2% ethanol for 24 hrs at 70% epiboly. **C**, treated with 2% ethanol for 24 hrs at 24 hpf. **D**, treated with 2% ethanol for 24 hrs at 2 dpf. **E**, treated with 2% ethanol for 24 hrs at 3 dpf. **F**, treated with 2% ethanol for 24 hrs at 4 dpf.
(TIF)

Figure S3 **Ethanol exposure later than 48 hpf had no effect on** ***isl1:GFP*** **expression. A–D**, *Tg(isl1:GFP)* larval at 7 dpf, dorsal views. **A**, is control. **B**, treated with 2% ethanol for 24 hrs at 2 dpf. **C**, treated with 2% ethanol for 24 hrs at 3 dpf. **D**, treated with 2% ethanol for 48 hrs at 4 dpf.
(TIF)

Figure S4 **Ethanol exposure had no apparent overall effect on post-mitotic neurons visualized by** ***elavl3:GFP*** **expression. A–C**, *Tg(elavl3:GFP)* larva at 5 dpf, DIC and fluorescent composite photomicrographs, dorsal views. **A**, is control. **B**, treated with 1% ethanol for 13 hrs at 35 hpf. **C**, treated with 2% ethanol for 13 hrs at 35 hpf.
(TIF)

Figure S5 **Ethanol exposure had no effect on the overall numbers of post-mitotic neurons in wild-type** ***Tg(elavl3:GFP)*** **embryos, while** ***med12^y82^*** **mutant embryos had fewer post-mitotic neurons that became further reduced after ethanol exposure. A–F**, lateral view of 36 hpf embryos. **A–C**, wild-type *Tg(elavl3:GFP)* embryos. **D–F**, *med12^y82^;Tg(elavl3:GFP)* embryos. **A, D**, controls. **B, E**, treated with 1% ethanol from dome stage to 24 hpf. **C, F**, treated with 2% ethanol for 24 hrs at 3 dpf. **G**, quantitation of GFP expression by real-time quantitative RT-PCR. GFP expression was normalized to β-actin as an internal control. Relative quantity was compared to control wild-type *Tg(elavl3:GFP)* embryos (HuC). The relative quantity of GFP in control wild-type *Tg(elavl3:GFP)* embryos was set to one, which is zero on the log scale. The relative expression of GFP trends higher in *med12^y82^* mutant (kto) embryos and in ethanol-treated embryos as shown by the red bars. Although the overall amount of GFP expression in *med12^y82^* mutant and ethanol-treated embryos appears to be somewhat reduced in panels A**–**F, when normalized to β-actin GFP expression is essentially equal or even increased relative to control wild-type embryos. This suggests that neither ethanol exposure nor *med12* mutation results in a relative reduction in post-mitotic neurons. Error bars represent 95% confidence levels.
(TIF)

Figure S6 **Early** ***pax2a:GFP*** **expression was similar in wild-type and** ***med12^y82^*** **embryos.** **A, B**, lateral view of 24 hpf embryos. **A**, wild-type *Tg(pax2a:GFP)* embryo. **B**, *med12^y82^;Tg(pax2a:GFP)* embryo.
(TIF)

Figure S7 ***Pax2a:GFP*** **expression was reduced in the hindbrain following ethanol exposure in wild-type, and is severely depleted in** ***med12^y82^*** **embryos. A−D**, lateral views of 60 hpf embryos. **A, B**, wild-type *Tg(pax2a:GFP)* embryos. **C, D**, *med12^y82^;Tg(pax2a:GFP)* embryos. **A, C**, controls. **B, D**, treated with 2% ethanol for 24 hrs at 24 hpf.
(TIF)
