# Supplementary figures and images for: Novel Oxytocin Gene Expression in the Hindbrain Is Induced by Alcohol Exposure: Transgenic Zebrafish Enable Visualization of Sensitive Neurons
Source: PLoS One. 2013 Jan 14;8(1):e53991. doi: 10.1371/journal.pone.0053991 (PMC3544674; doi:10.1371/journal.pone.0053991)

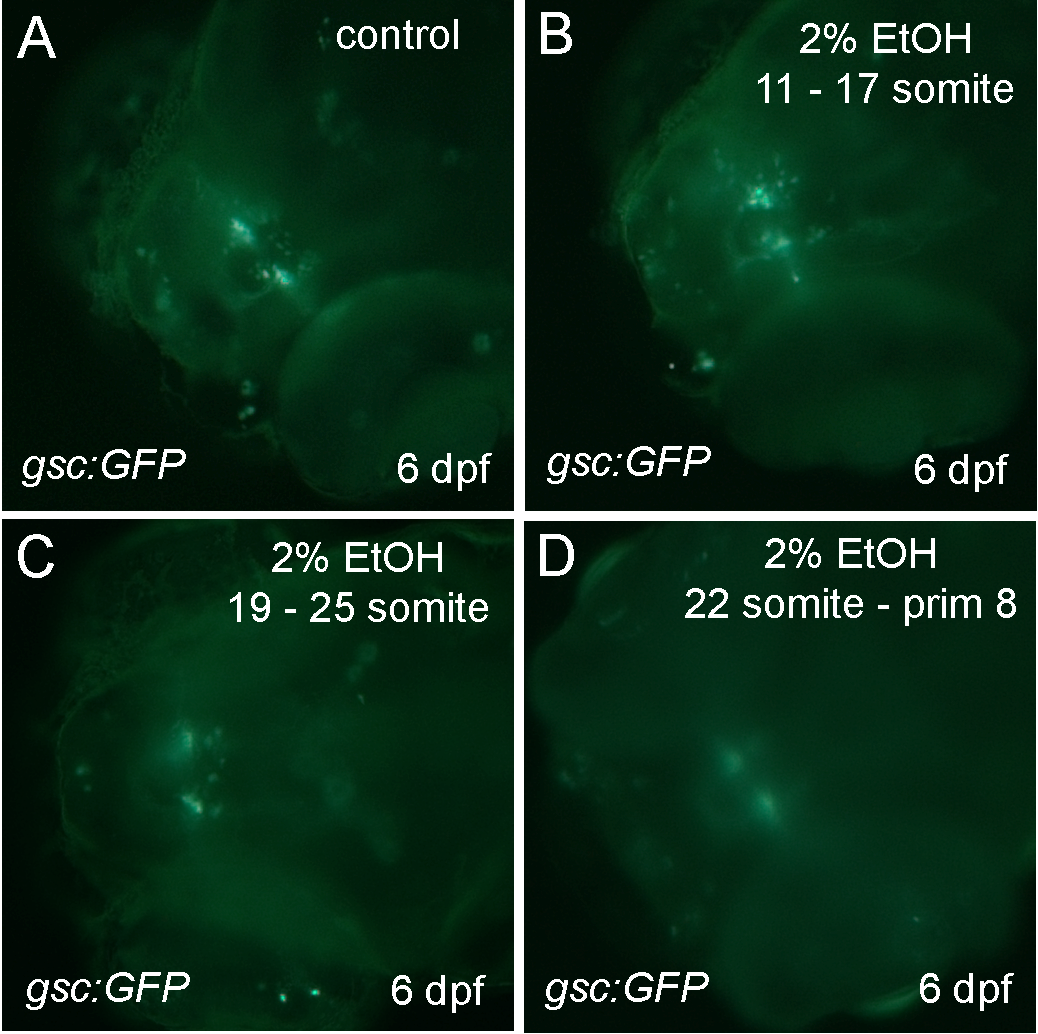

Supplement: Figure S1 — Ethanol exposure during the predicted time period of initial differentiation of gsc -expressing cells had no effect on gsc:GFP expression. A–D, Tg(gsc:GFP) larval at 6 dpf, dorsal views. A, is control. B, treated with 2% ethanol from 11 to 17 somites stage. C, treated with 2% ethanol from 19 to 25 somites stage. D, treated with 2% ethanol from 22 somites to prim8 stage. (TIF) [file pone.0053991.s001.tif]

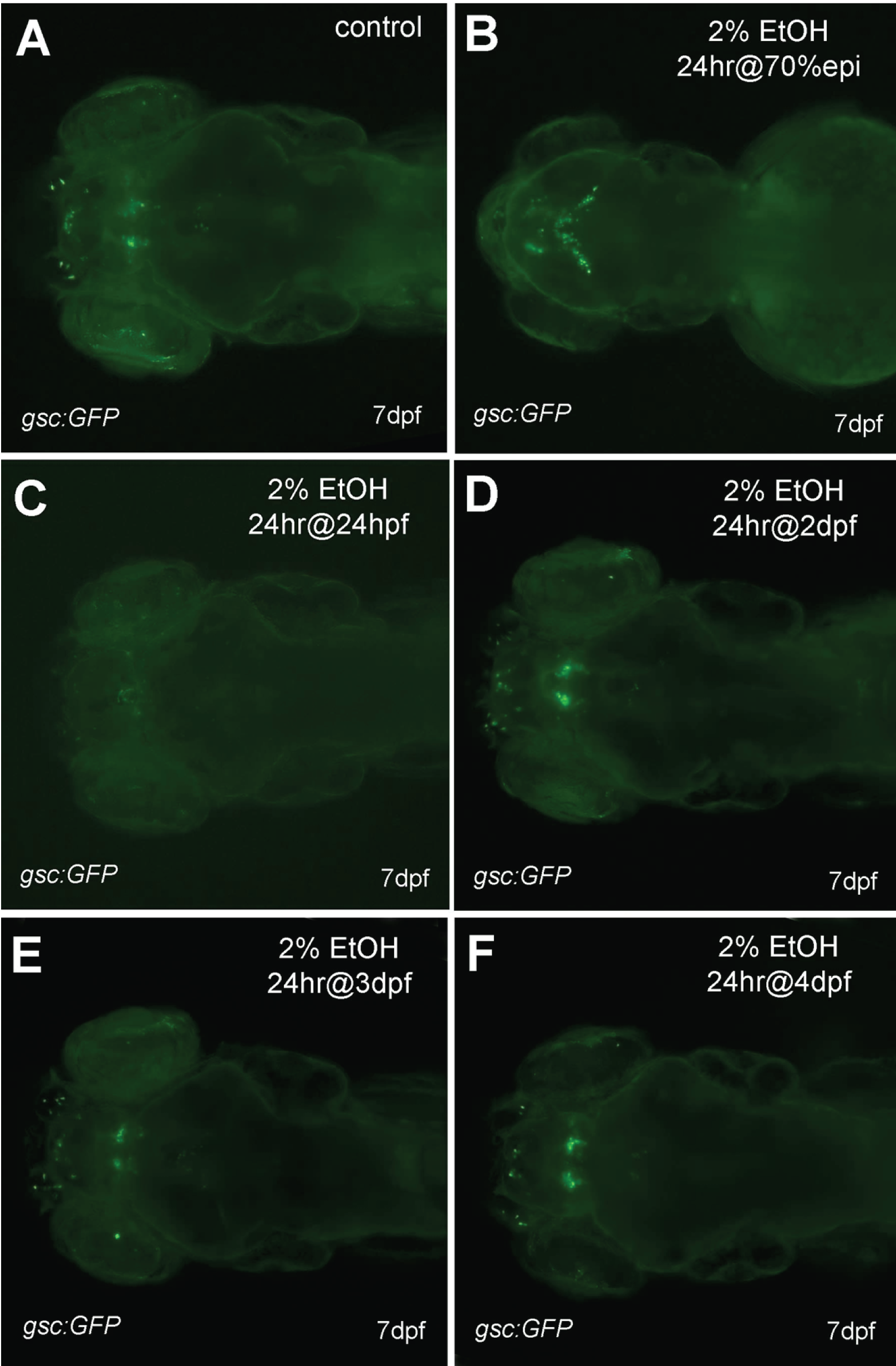

Supplement: Figure S2 — Ethanol exposure later than 48 hpf had no effect on gsc:GFP expression. A–F, Tg(gsc:GFP) larval at 7 dpf, dorsal views. A, is control. B, treated with 2% ethanol for 24 hrs at 70% epiboly. C, treated with 2% ethanol for 24 hrs at 24 hpf. D, treated with 2% ethanol for 24 hrs at 2 dpf. E, treated with 2% ethanol for 24 hrs at 3 dpf. F, treated with 2% ethanol for 24 hrs at 4 dpf. (TIF) [file pone.0053991.s002.tif]

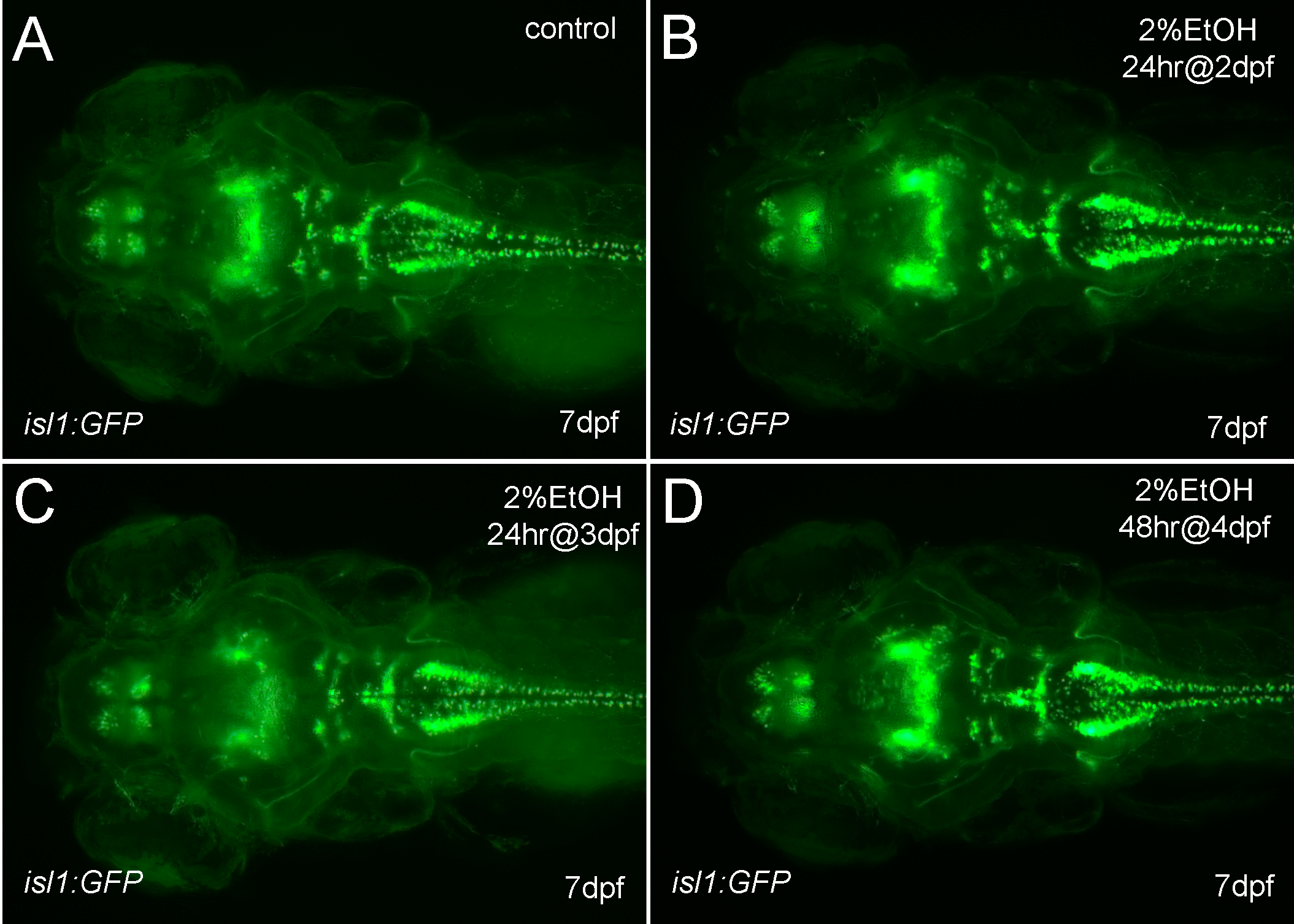

Supplement: Figure S3 — Ethanol exposure later than 48 hpf had no effect on isl1:GFP expression. A–D, Tg(isl1:GFP) larval at 7 dpf, dorsal views. A, is control. B, treated with 2% ethanol for 24 hrs at 2 dpf. C, treated with 2% ethanol for 24 hrs at 3 dpf. D, treated with 2% ethanol for 48 hrs at 4 dpf. (TIF) [file pone.0053991.s003.tif]

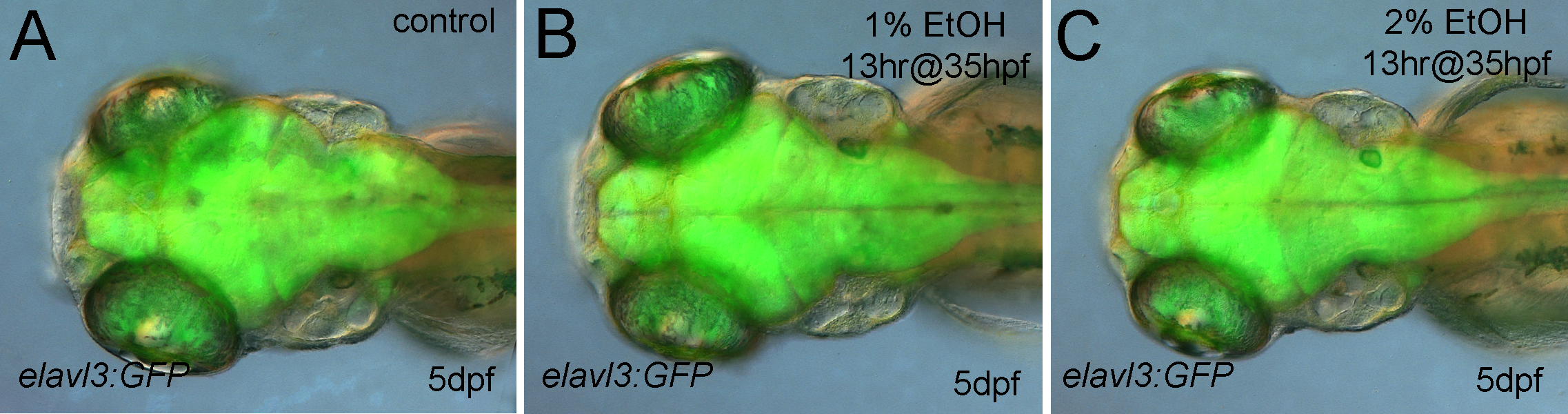

Supplement: Figure S4 — Ethanol exposure had no apparent overall effect on post-mitotic neurons visualized by elavl3:GFP expression. A–C, Tg(elavl3:GFP) larva at 5 dpf, DIC and fluorescent composite photomicrographs, dorsal views. A, is control. B, treated with 1% ethanol for 13 hrs at 35 hpf. C, treated with 2% ethanol for 13 hrs at 35 hpf. (TIF) [file pone.0053991.s004.tif]

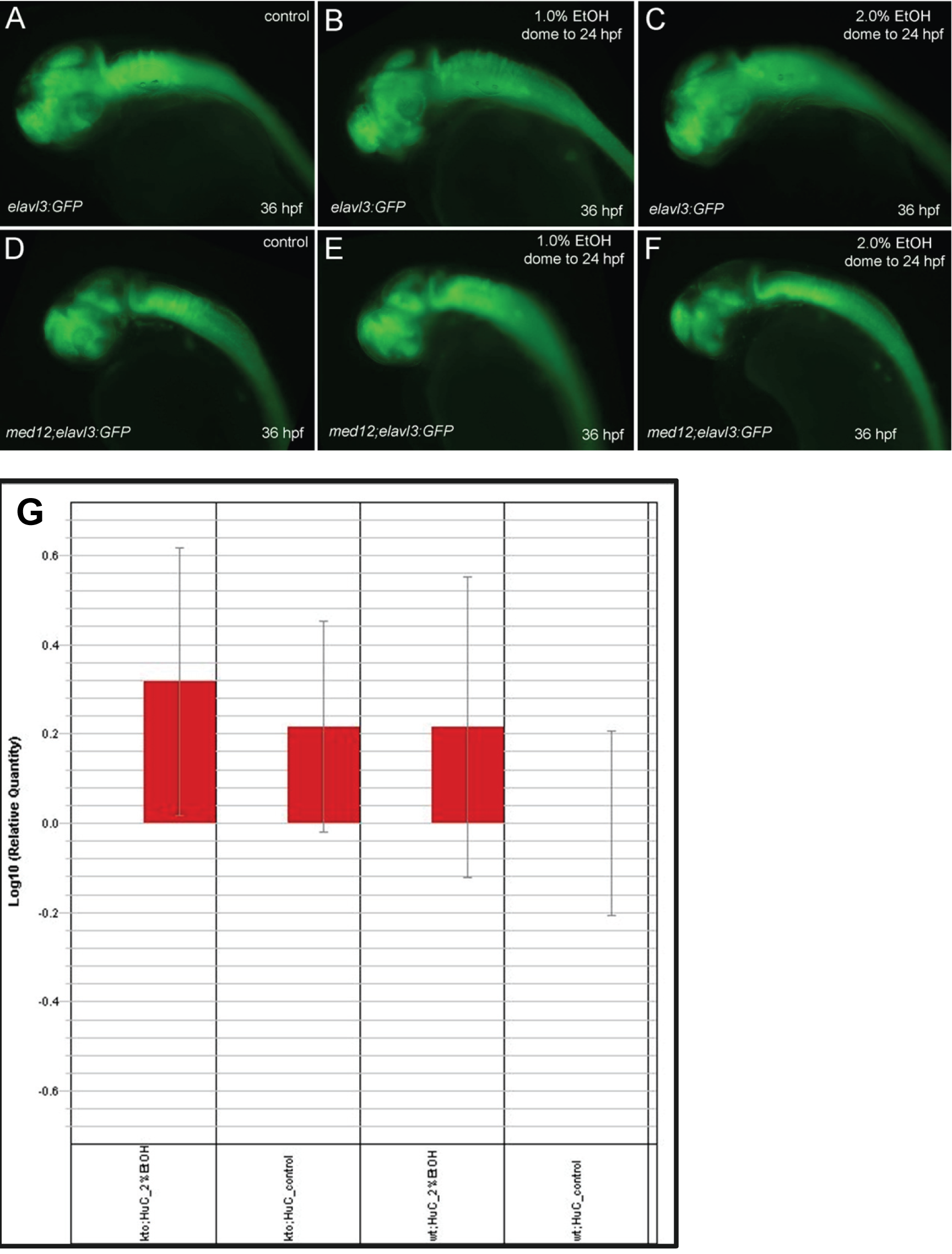

Supplement: Figure S5 — Ethanol exposure had no effect on the overall numbers of post-mitotic neurons in wild-type Tg(elavl3:GFP) embryos, while med12y82 mutant embryos had fewer post-mitotic neurons that became further reduced after ethanol exposure. A–F, lateral view of 36 hpf embryos. A–C, wild-type Tg(elavl3:GFP) embryos. D–F, med12y82;Tg(elavl3:GFP) embryos. A, D, controls. B, E, treated with 1% ethanol from dome stage to 24 hpf. C, F, treated with 2% ethanol for 24 hrs at 3 dpf. G, quantitation of GFP expression by real-time quantitative RT-PCR. GFP expression was normalized to β-actin as an internal control. Relative quantity was compared to control wild-type Tg(elavl3:GFP) embryos (HuC). The relative quantity of GFP in control wild-type Tg(elavl3:GFP) embryos was set to one, which is zero on the log scale. The relative expression of GFP trends higher in med12y82 mutant (kto) embryos and in ethanol-treated embryos as shown by the red bars. Although the overall amount of GFP expression in med12y82 mutant and ethanol-treated embryos appears to be somewhat reduced in panels A–F, when normalized to β-actin GFP expression is essentially equal or even increased relative to control wild-type embryos. This suggests that neither ethanol exposure nor med12 mutation results in a relative reduction in post-mitotic neurons. Error bars represent 95% confidence levels. (TIF) [file pone.0053991.s005.tif]

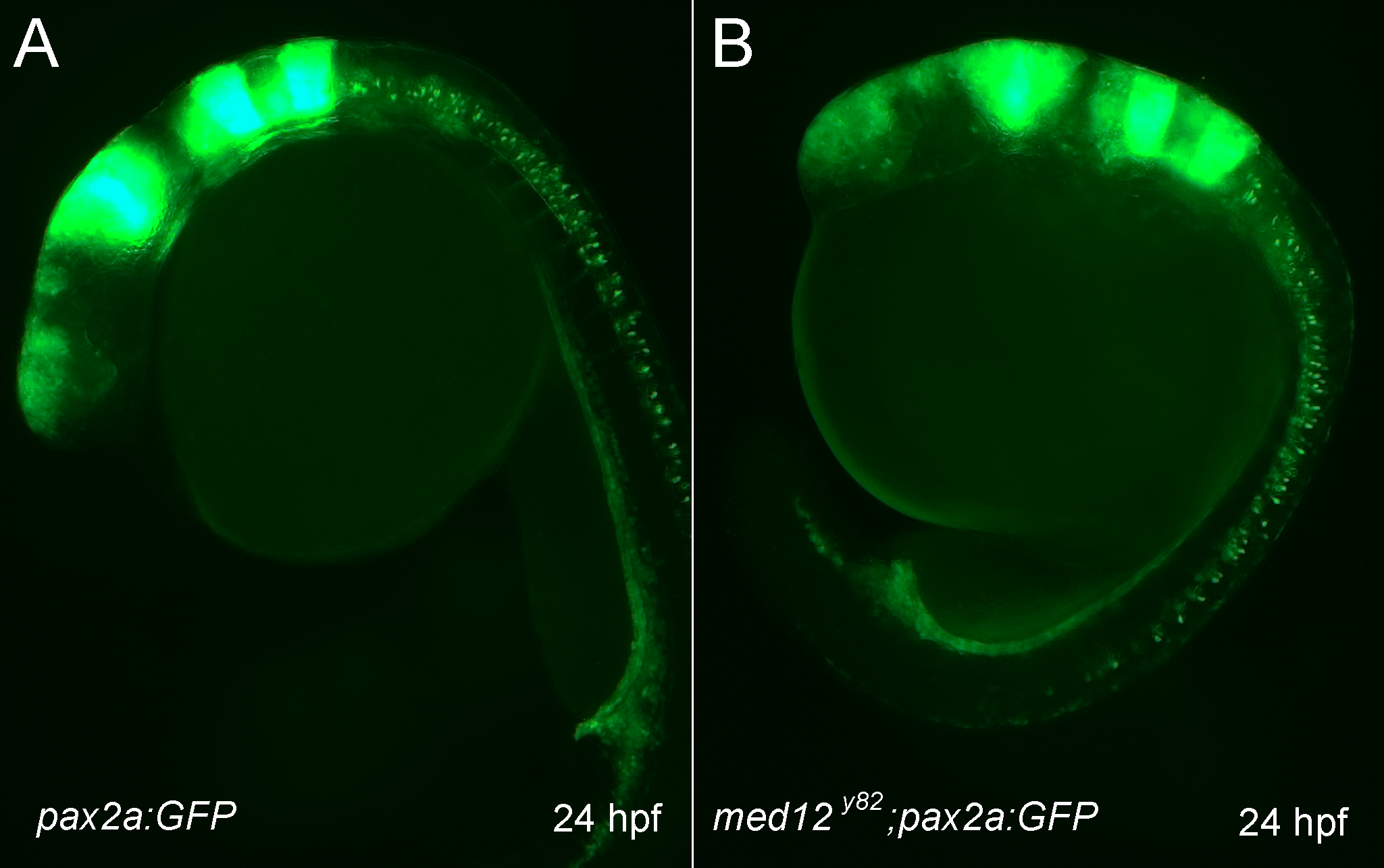

Supplement: Figure S6 — Early pax2a:GFP expression was similar in wild-type and med12y82 embryos. A, B, lateral view of 24 hpf embryos. A, wild-type Tg(pax2a:GFP) embryo. B, med12y82;Tg(pax2a:GFP) embryo. (TIF) [file pone.0053991.s006.tif]

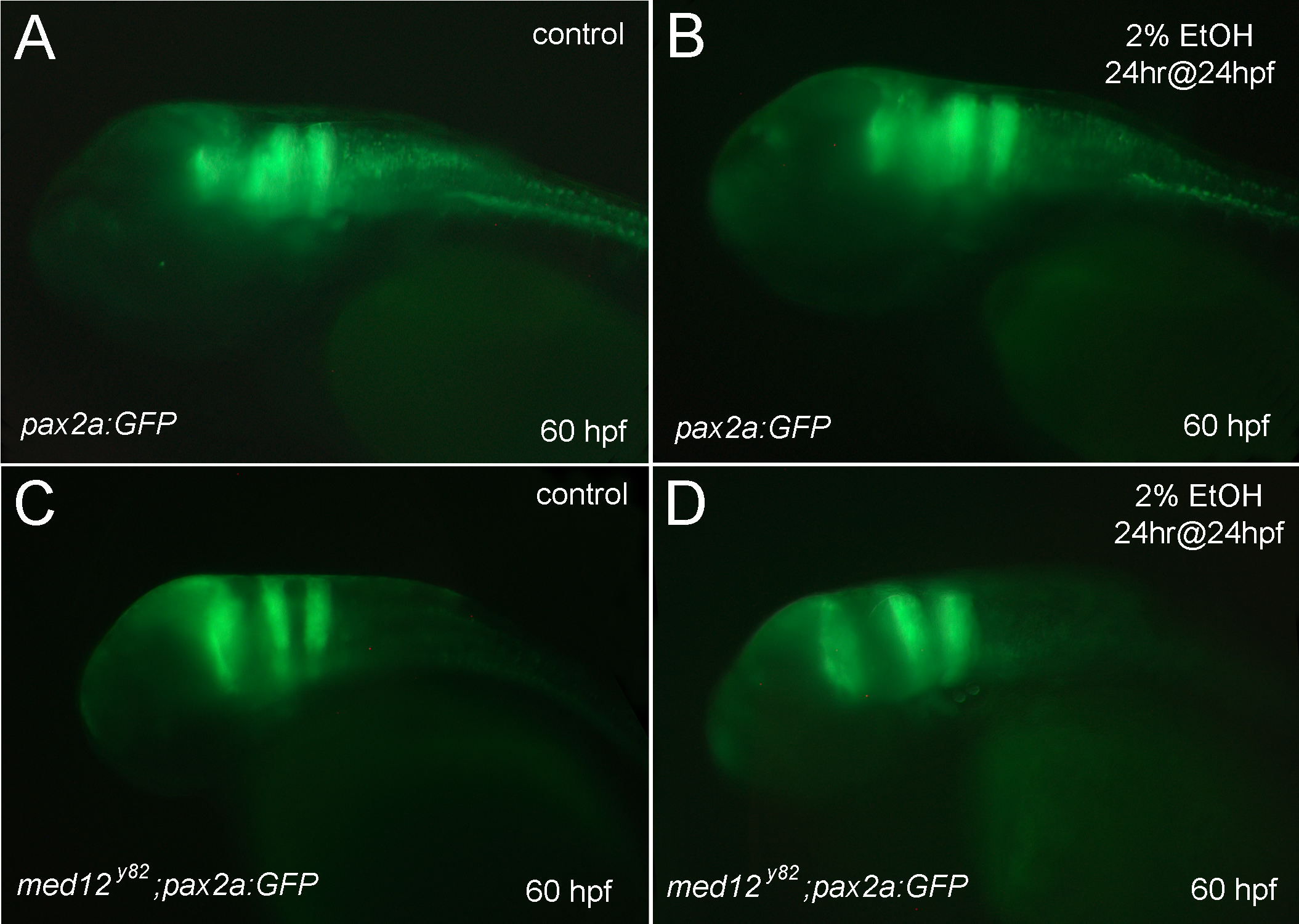

Supplement: Figure S7 — Pax2a:GFP expression was reduced in the hindbrain following ethanol exposure in wild-type, and is severely depleted in med12y82 embryos. A−D, lateral views of 60 hpf embryos. A, B, wild-type Tg(pax2a:GFP) embryos. C, D, med12y82;Tg(pax2a:GFP) embryos. A, C, controls. B, D, treated with 2% ethanol for 24 hrs at 24 hpf. (TIF) [file pone.0053991.s007.tif]
